# The expression profile and clinical application value of hsa_circ_0016148 in head and neck squamous cell carcinoma

**DOI:** 10.1002/jcla.23997

**Published:** 2021-09-30

**Authors:** Zhisen Shen, Liuqian Wang, Dong Ye

**Affiliations:** ^1^ Department of Otorhinolaryngology Head and Neck Surgery Ningbo Medical Center Lihuili Hospital Ningbo China; ^2^ Department of Otorhinolaryngology Head and Neck Surgery Lihuili Hospital Affiliated to Ningbo University Ningbo China; ^3^ Medical School of Ningbo University Ningbo China

**Keywords:** circular RNA, diagnosis, head and neck squamous cell carcinoma, hsa_circ_0016148, prognosis

## Abstract

**Background:**

Dysregulated circular RNAs (circRNAs) are involved in human cancers and may be used as biomarkers with the potential of clinical application. However, little is known regarding the mechanism of circRNAs and their clinical application value in head and neck squamous cell carcinoma (HNSCC).

**Methods:**

In the current study, we established the profile of circRNAs in HNSCC using microarray and then measured the expression of hsa_circ_0016148 in 137 paired HNSCC tissues by qRT‐PCR technique, analyzed the relationship between hsa_circ_0016148 and clinicopathological data, and investigated its diagnostic and prognostic value. The hsa_circ_0016148‐miRNA‐mRNA interaction network was predicted and constructed by Cytoscape.

**Results:**

Our study showed a circRNA expression profile and confirmed downregulated hsa_circ_0016148 in HNSCC tissues (*p* = 2.64E‐35). The hsa_circ_0016148 expression is remarkably correlated with lymph node metastasis (*p* = 0.001) and clinical stage (*p* = 0.029). Then, the area under the receiver characteristic curve (AUC) was 0.912 with 92% of sensitivity and 86.9% specificity, respectively. Besides, our study demonstrated that lower‐expressed hsa_circ_0016148 could independently predict poorer overall survival of HNSCC patients (hazard ratio [HR] = 0.456; 95% confidence interval [CI] = 0.265–0.784; *p* = 0.005). The hsa_circ_0016148‐miRNA‐mRNA interaction network was constructed, which included a total of nine targeted miRNAs.

**Conclusion:**

Taken together, our results revealed that hsa_circ_0016148 might play a critical role in HNSCC tumorigenesis and may serve as an indicator with the potential of diagnosis and prognosis for HNSCC.

## Graphical abstract

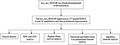

## 1 INTRODUCTION

Head and neck cancers are the seventh most common forms of cancer worldwide, encompassing a wide range of tumors arising from numerous anatomic subsites including the nasal cavity, paranasal sinuses, nasopharynx, oral cavity, lips, oropharynx, hypopharynx, and larynx.^1^ Head and neck squamous cell carcinoma (HNSCC) is the most frequent histological type, accounting for more than 90% of head and neck cancers.^2^, ^3^ The incidence of HNSCC is approximately 1,000,000 patients diagnosed worldwide, and about half cases were dead annually.^4^ The high‐risk factors for HNSCC include upper aerodigestive tract mucosa exposure to carcinogens such as tobacco, alcohol, and human papillomavirus (HPV), with a multiplicative effect.^5^, ^6^ While the use of tobacco is decreasing around the world, there is a recent upsurge in the incidence of HPV‐related HNSCC.^7^ Despite improvements in radiation technology and techniques, better surgical options for organ preservation, and multidisciplinary treatment modalities, HNSCC patients continue to have poor survival rates due to local recurrence and distant metastasis, particularly in advanced patients.^8^ Recently, immune‐checkpoint inhibitors nivolumab and pembrolizumab have been approved for treatment of platinum chemotherapy‐failed HNSCC patients with recurrent or metastatic. This treatment has provided significantly better outcomes, but only a small proportion of patients benefit from it. The 5‐year survival rate is still only 50% and has slightly improved in the last two decades.^2^ The current plight is partly due to the lack of accurate and reliable tools for early diagnosis and prognosis prediction of HNSCC.^9^ Therefore, identifying an effective and specific biomarker is urgent for severity assessment and improvement of personalized medical treatment for patients with HNSCC.

In the beginning, circular RNAs (circRNAs) are recognized as a class of noncoding RNAs that are covalently bonded to form a special closed‐loop structure without 5′ caps and 3′ tails.^10^ However, recent studies have shown that some circRNAs could also be translated, which might be driven by a single N6‐methyladenosine residue.^11^, ^12^ CircRNAs are considered as abundant, stable, and conserved across evolutions, and many play important biological functions by acting as microRNA (miRNA) sponges that regulate protein function or by being translated themselves.^13^ In addition, circRNAs can modulate several important physiological processes through interacting with RNA‐binding protein, such as apoptosis.^14^ Evidence is increasing that circRNAs are involved in several diseases including cancers.^13^, ^15^ One recent study by Zhang and his colleagues showed that circular RNA circ_0001287 inhibited the proliferation, metastasis, and radiosensitivity of non‐small cell lung cancer cells by sponging miR‐21.^16^ Hao et al. reported that circDCUN1D4 suppressed tumor metastasis and glycolysis in lung adenocarcinoma by stabilizing TXNIP expression. Additionally, due to its characteristics of abundance, diversity, stability, and disease‐specific exhibition, circRNA was considered as ideal biomarker for auxiliary diagnosis and prognosis estimation of disease.^17^, ^18^ Nevertheless, to the best of our knowledge, the mechanism of circRNAs in HNSCC is still fairly elusive.

In the current study, a circRNA expression profile was constructed from eighteen paired HNSCC samples and corresponding normal tissues using a microarray platform. circRNA‐ hsa_circ_0016148 was found downregulated in the microarray and selected for further study. The gene of hsa_circ_0016148 is located at chromosome chr1:204410598–204411761. Its spliced sequence length is 201 nt; the associated gene symbol is phosphatidylinositol‐4‐phosphate 3‐kinase catalytic subunit type 2 beta (*PIK3C2B*). We believe that this is the first time hsa_circ_0016148 expression in HNSCC tissues and normal controls has been investigated or that the relationship with clinicopathological factors of patients with HNSCC has been evaluated. Moreover, we explored its potential diagnostic and prognostic value and regulatory mechanisms for HNSCC. Potential biological functions were further predicted and annotated by bioinformatics analysis.

## 2 MATERIALS AND METHODS

### 2.1 Sample collection and clinicopathological data

A total of 137 HNSCC patients with a histopathological diagnosis (127 male and 10 female, age range 39‑75 years; average age 61.5 years) were recruited from the Affiliated Tumor Hospital of Xiangya Medical School (Changsha, China) and the Ningbo Medical Centre Lihuili Hospital (Ningbo, China), from February 2014 through November 2019. A circRNA microarray was performed using eighteen paired HNSCC specimens and corresponding normal tissues to investigate the dysregulated circRNAs. None of the patients had received radiation, chemotherapy, or other cancer treatment before samples were taken. After the surgical resection, tissue samples were frozen immediately in liquid nitrogen and stored at −80°C in an RNA‐fixer reagent (Bioteke) until RNA extraction. The clinicopathological characteristics were abstracted from medical records, and tumor clinical stages and histological grades were assessed according to the 8th TNM staging system of Edition American Joint Committee on Cancer (AJCC).^19^ During the observation period, 11 patients were censored and 61 patients died. Overall survival (OS) time was measured from the date of diagnosis to death from any cause. The human research ethics approval from Affiliated Tumor Hospital of Xiangya Medical School and Ningbo Medical Centre Lihuili Hospital is obtained before the study begins. Informed consents were signed by all included patients.

### 2.2 Total RNA extraction and quantitative real‐time PCR assay

Following the manufacturer's instructions, we extracted total RNA from tissues using TRIzol reagent (Invitrogen). Then, cDNA was reverse‐transcribed using the GoScript Reverse Transcription (RT) System (Promega). The primers for the amplified sequences of hsa_circ_0016148 were: forward, 5′‐TCTTCAAGGGAATCCTCCGC‐3′, and reverse, 5′‐CCTTAACCAGCAGACTGGGG‐3′. *GAPDH* was simultaneously amplified as internal control.^20^ RT‐PCR was performed using the RocheLightCycler 480II System (Roche). The PCR was conducted according to the following conditions: denaturation at 95°C for 10 min, then 40 cycles at 95°C for 20 s, 60°C for 30 s, and 72°C for 40 s. The quantification cycle (Cq) values of hsa_circ_0016148 and *GAPDH* were recorded. The relative quantification level of hsa_circ_0016148 was calculated by the ΔCq method using *GAPDH* as a reference gene. All data were presented as means ± SD (standard deviation) through at least three independent experiments.

### 2.3 Diagnostic value analysis

We generated a receiver operator curve (ROC) and calculated the area under curve (AUC) to assess the diagnostic efficacy of hsa_circ_0016148.^21^ The maximum Youden index was established to identify the optimal cut‐off value of ROC.

### 2.4 Survival analysis

According to the optimal cut‐off value of ROC, HNSCC patients were divided into high and low hsa_circ_0016148 groups. Then, Kaplan–Meier analysis was performed between these two groups. Univariate Cox regression analysis was used to identify potential prognostic factors, which were then involved in the multivariate Cox regression analysis to evaluate the independent risk factors associated with overall survival of HNSCC.

### 2.5 Finding the potential target miRNAs of hsa_circ_0016148 and ceRNA network analysis with bioinformatics

First, we used the online circinteractome database (https://circinteractome.irp.nia.nih.gov/index.html) to predict miRNA targets of hsa_circ_0016148. Then, the target genes of these miRNAs were predicted from the TARBASE v.8 and miRTARBASE databases. Cytoscape software was used to construct the ternary circRNA‐miRNA‐mRNA interaction network. Finally, GO enrichment analysis was conducted for all target genes using org. Hs.eg.db and the clusterProfiler package in R 3.6.0 software.

### 2.6 Statistical analyses

SPSS version 18.0 (SPSS Inc.) was used for all statistical analysis. The comparison of hsa_circ_0016148 expression was made using paired sample Student's *t* test between HNSCC samples and controls. The association between hsa_circ_0016148 and clinical parameters was explored by independent sample Student's t test. We considered estimates to be statistically significant if the *p* value was less than 0.05 with a two‐tailed test.

## 3 RESULTS

### 3.1 Comparison of expression of hsa_circ_0016148 in HNSCC

**FIGURE 1:**
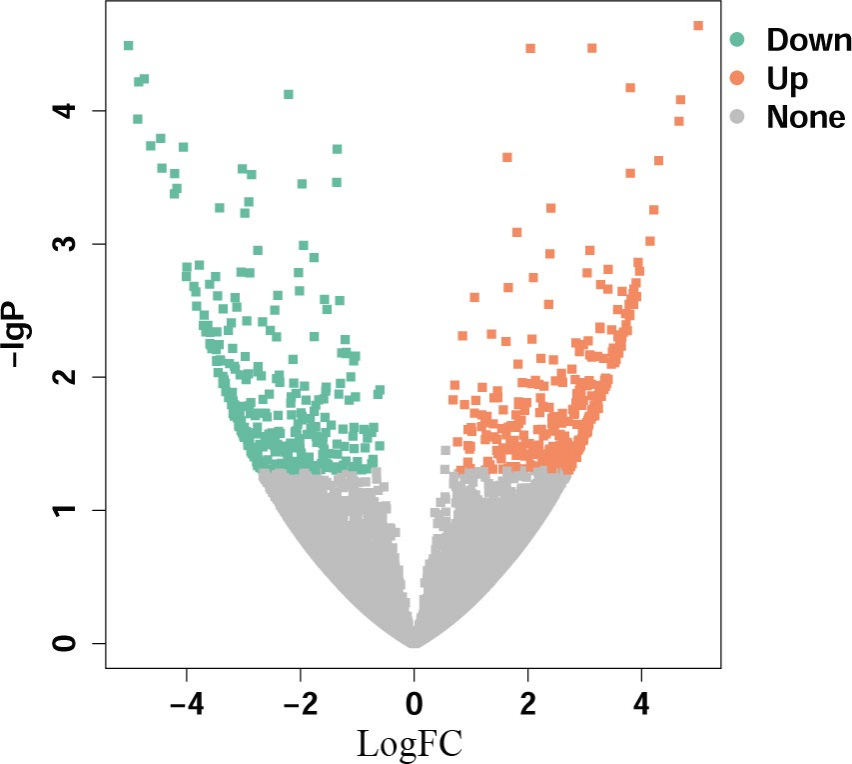
Volcano plot of circRNA microarray in head and neck squamous cell carcinoma (HNSCC). Log FC >3, *p* < 0.05

**FIGURE 2:**
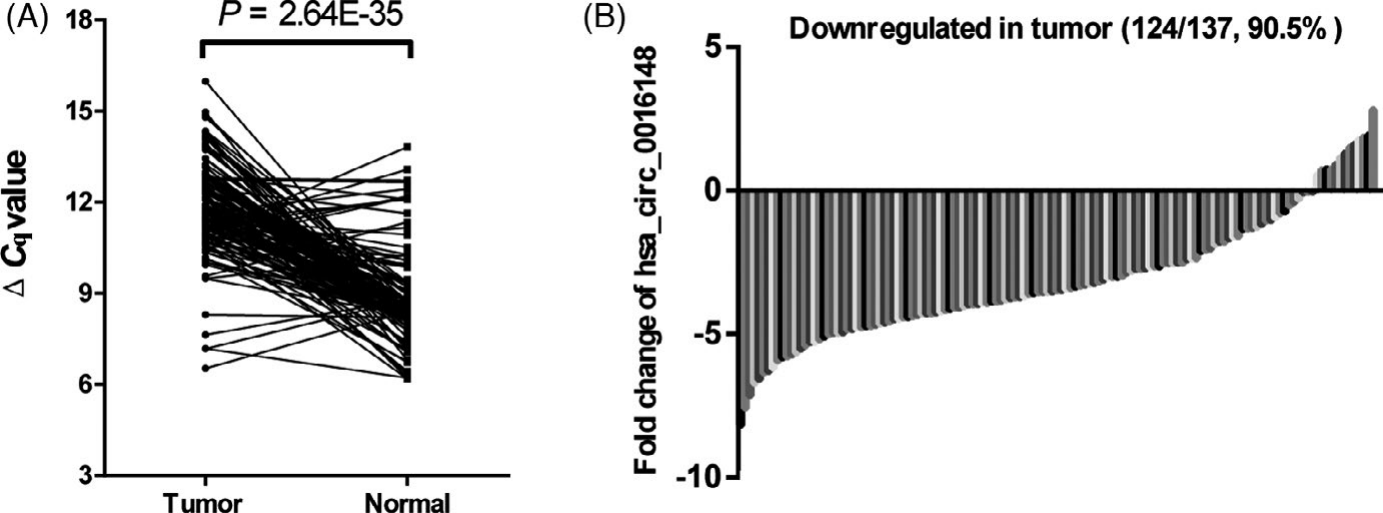
hsa_circ_0016148 expression levels in 137 paired head and neck squamous cell carcinoma (HNSCC) tissues and corresponding normal tissues. (A) the expression level of hsa_circ_0016148 was significantly downregulated in HNSCC. (B) hsa_circ_0016148 expression levels were downregulated in 90.5% (124/137) of HNSCC tissues compared with adjacent normal tissues

In this study, we established a circRNA expression profile from eighteen paired HNSCC specimens and adjacent normal tissues using a microarray platform. The volcano plot is shown in Figure 1. Hsa_circ_0016148 was found downregulated in the microarray and selected for further study. We confirmed hsa_circ_0016148 expression levels in 137 paired HNSCC tissues and corresponding normal tissues by quantitative real‐time polymerase chain reaction. The sequence of qRT‐PCR product was identical to that from circBase (http://circbase.org/). We found that hsa_circ_0016148 expression in HNSCC tissues was remarkably decreased than that in adjacent normal tissues (*p* = 2.64E‐35, Figure 2A). The expression of hsa_circ_0016148 was downregulated in 90.5% (124/137) of HNSCC tissues compared with the corresponding non‐tumorous tissues (Figure 2B).

### 3.2 Association between hsa_circ_0016148 expression and clinicopathological parameters in HNSCC patients

Subsequently, we analyzed the association between hsa_circ_0016148 expression level and clinicopathological characteristics, including age, gender, smoking behavior, alcohol behavior, tumor site, differentiation grade, tumor stage, lymph node metastasis, and clinical stage. As shown in Table 1, low hsa_circ_0016148 expression in HNSCC tissues was significantly associated with lymphatic metastasis (*p* = 0.001) and clinical stage (*p* = 0.029). The rest of clinicopathological characteristics showed slight association between hsa_circ_0016148 expression levels in HNSCC patients.

**TABLE 1:** Association of hsa_circ_0016148 with clinicopathological features of HNSCC patients

Characteristics	n	Mean ± SD	*p* Value
Gender			
Female	10	11.891 ± 1.438	0.961
Male	127	11.867 ± 1.521	
Age			
<60 years	63	11.632 ± 1.778	0.102
>60 years	74	12.069 ± 1.213	
Smoking behavior			
No	69	11.849 ± 1.486	0.881
Yes	68	11.888 ± 1.544	
Alcohol behavior			
No	45	11.528 ± 1.659	0.064
Yes	92	12.035 ± 1.411	
Tumor site			
Oral cavity/oropharynx	29	11.682 ± 1.918	0.456
Larynx/hypopharynx	108	11.918 ± 1.386	
Histologic grade			
Well	39	11.959 ± 1.570	0.659
Moderately/poorly	98	11.832 ± 1.492	
Tumor invasion			
Tis/T1/T2	80	11.725 ± 1.466	0.188
T3/T4	57	12.070 ± 1.559	
Lymphatic metastasis			
No	84	11.542 ± 1.503	**0.001**
Yes	53	12.386 ± 1.382	
Clinical stage			
I + II	69	11.590 ± 1.456	**0.029**
III + IV	68	12.150 ± 1.521	

Note

*p* Value in bold indicates statistical significance.

Abbreviations: HNSCC, head and neck squamous cell carcinoma; n, number; SD, standard deviations.

### 3.3 The receiver operating characteristic (ROC) curve of hsa_circ_0016148 in HNSCC

**FIGURE 3:**
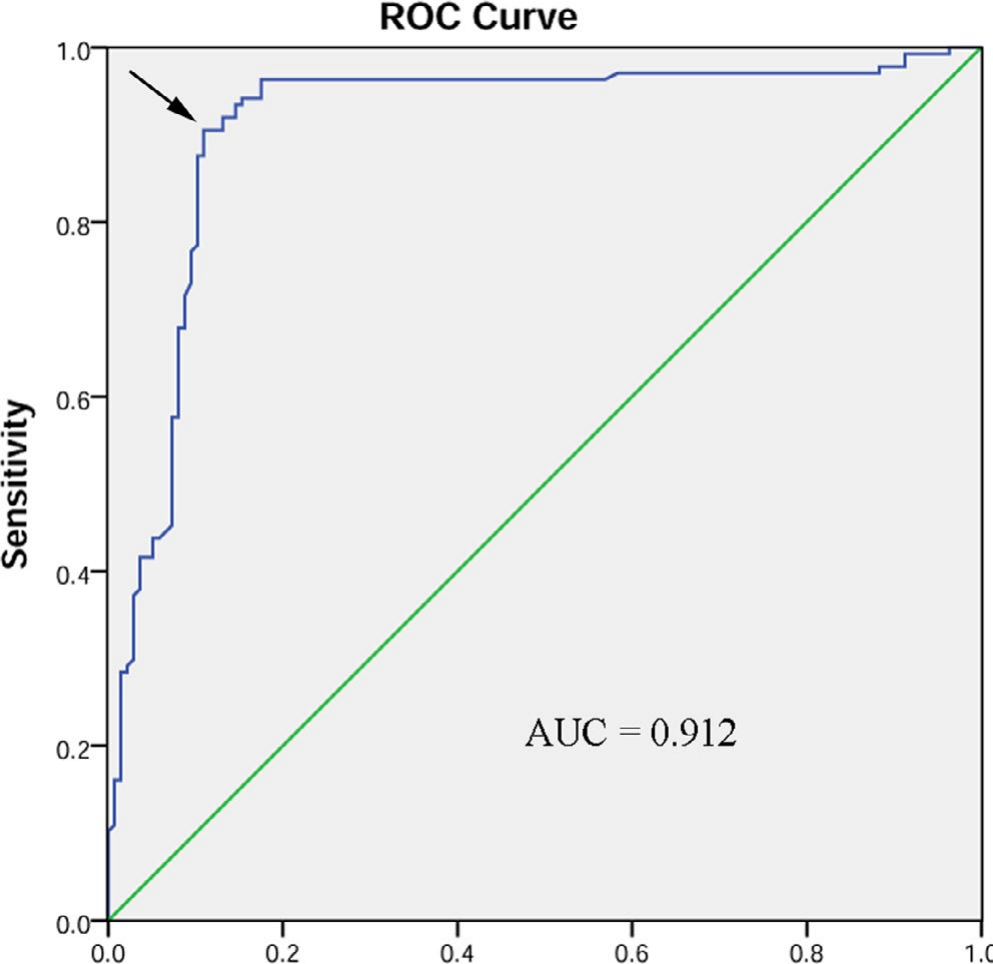
Receiver operating characteristic (ROC) curve. The cut‐off point was defined as the maximum Youden index, highlighted by the arrow in the figure

The potential diagnostic value of hsa_circ_0016148 was identified by ROC curve in HNSCC patients. The value of Youden index was calculated by the following formula: sensitivity + specificity‐1, and the maximum Youden index was defined as a cut‐off point. In our study, the area under the ROC curve (AUC) was 0.912 at a cut‐off value of 11.715 with 92% sensitivity and 86.9% specificity, respectively (Figure 3).

### 3.4 The prognostic potential of hsa_circ_0016148 in HNSCC

**FIGURE 4:**
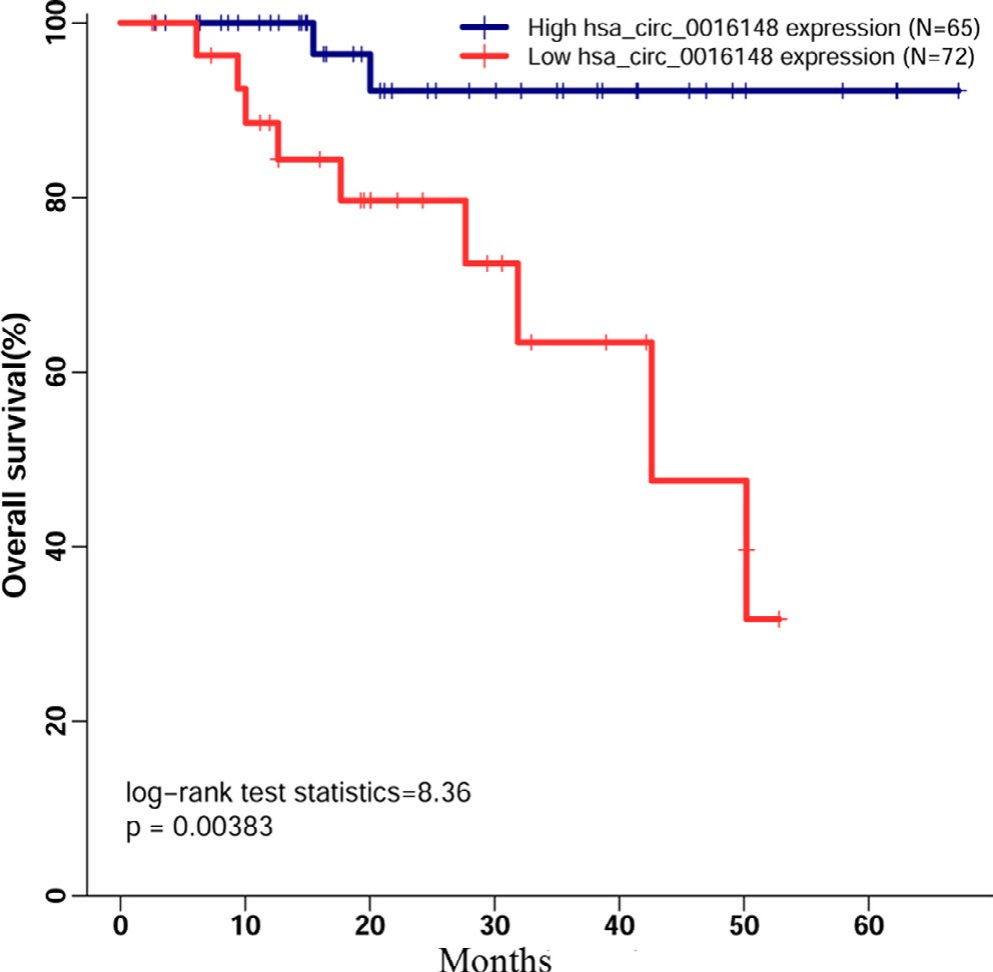
Association between hsa_circ_0016148 expression and overall survival in head and neck squamous cell carcinoma (HNSCC). Low hsa_circ_0016148 expression is associated with poor overall survival (OS) in HNSCC patients

Firstly, we applied the maximum Youden index and cut‐off value of ROC for further survival analysis. A total of 65 patients were grouped into high‐expression group, while the rest of HNSCC patients were assigned to the low‐expression group. The prediction value of hsa_circ_0016148 was investigated by Kaplan–Meier survival analysis in HNSCC. The Kaplan–Meier survival analysis revealed that low‐expression levels of hsa_circ_0016148 were significantly associated with poor outcome for HNSCC patients (Figure 4, log‐rank *p* = 0.004). Subsequently, we performed univariate Cox proportional hazards analysis to quantify the prognostic value of hsa_circ_0016148, and our results showed that 50.6% patients with low expression were at risk for death compared to patients with high expression of hsa_circ_0016148 (hazard ratio [HR] = 0.494; 95% confidence interval [CI] = 0.289–0.844; *p* = 0.010). To avoid the effect of lymphatic metastasis to survival, we performed a multivariate Cox proportional hazard analysis to identify the independent prediction capability of hsa_circ_0016148. The results indicated that low expression of hsa_circ_0016148 (HR = 0.456; 95% CI = 0.265–0.784; *p* = 0.005) and lymphatic metastasis (HR = 1.933; 95% CI = 1.141–3.275; *p* = 0.014) could be served as independent predictive biomarkers of OS in HNSCC patients (Table 2).

**TABLE 2:** Univariate and multivariate Cox regression analyses of overall survival in HNSCC patients

Variables	Univariate analysis			Multivariate analysis		
	HR	95% CI	*p*	HR	95% CI	*p*
Age (≥60 years vs. <60 years)	1.039	0.620–1.741	0.885			
Gender (female vs. male)	0.915	0.364–2.301	0.850			
Smoking behavior (Yes vs. No)	1.280	0.760–2.156	0.353			
Alcohol behavior (Yes vs. No)	0.924	0.527–1.618	0.781			
Tumor site (oral cavity/oropharynx vs. larynx/hypopharynx)	0.596	0.299–1.186	0.140			
Histologic grade (moderately/poorly vs. well)	1.529	0.905–2.582	0.112			
Tumor invasion (T3/T4 vs. Tis/T1/T2)	1.631	0.977–2.723	0.061			
Lymphatic metastasis (positive vs. negative)	1.742	1.036–2.929	**0.036**	1.933	1.141–3.275	**0.014**
Pathologic stage (III/IV vs. I/II)	1.636	0.986–2.715	0.057			
hsa_circ_0016148 expression (high vs. low)	0.494	0.289–0.844	**0.010**	0.456	0.265–0.784	**0.005**

Note

*p* Value in bold indicates statistical significance.

Abbreviations: CI, confidence interval; HR, hazard ratio; HNSCC, head and neck squamous cell carcinoma.

### 3.5 Construction of hsa_circ_0016148‐based regulatory network in HNSCC

**FIGURE 5:**
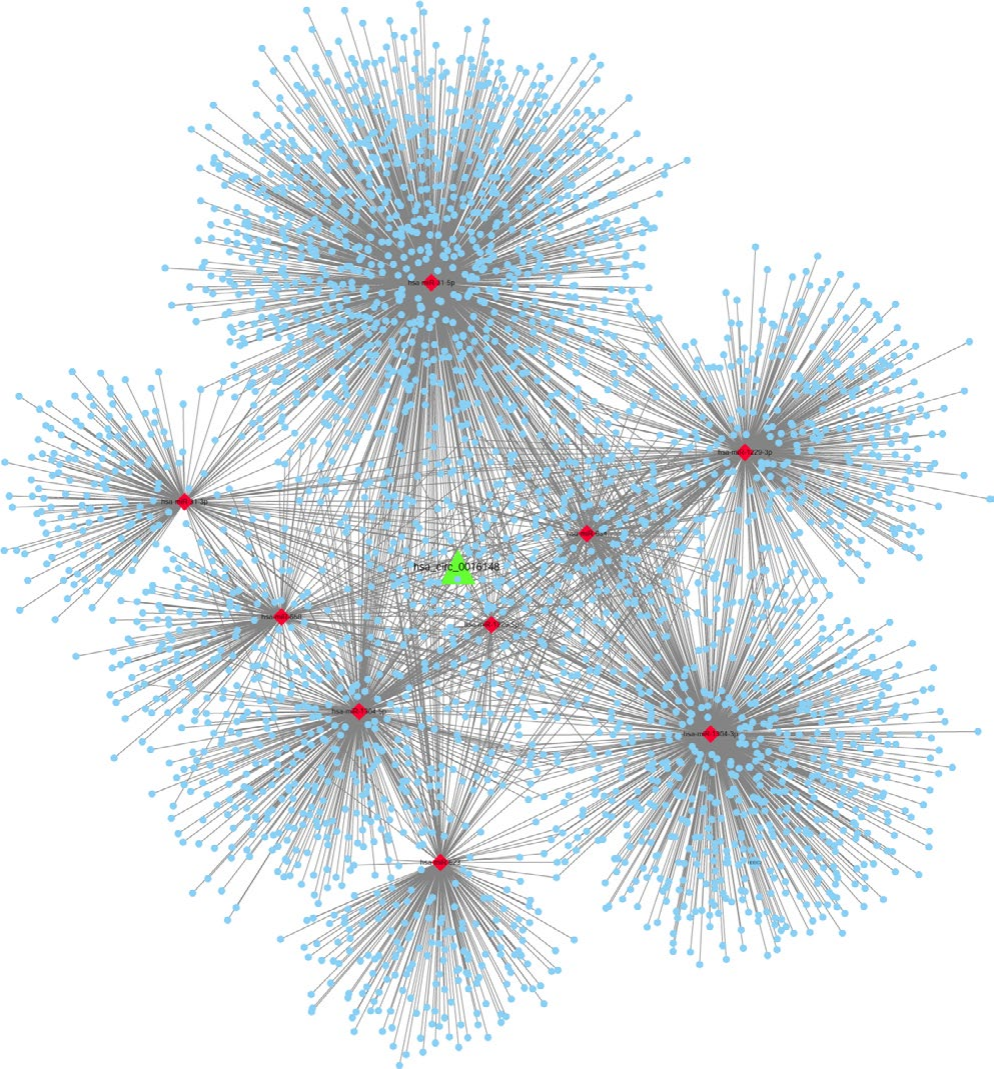
hsa_circ_0016148‐mediated circRNA‐miRNA‐mRNA regulatory network in head and neck squamous cell carcinoma. CircInteractome software showed that hsa_circ_0016148 is closely related to nine miRNAs (hsa‐miR‐31‐3p, hsa‐miR‐31‐5p, hsa‐miR‐623, hsa‐miR‐634, hsa‐miR‐658, hsa‐miR‐1229‐3p, hsa‐miR‐1229‐5p, hsa‐miR‐1304‐3p, and hsa‐miR‐1304‐5p). The target genes of these nine miRNAs were predicted from the TARBASE v.8 and miRTARBASE databases

**FIGURE 6:**
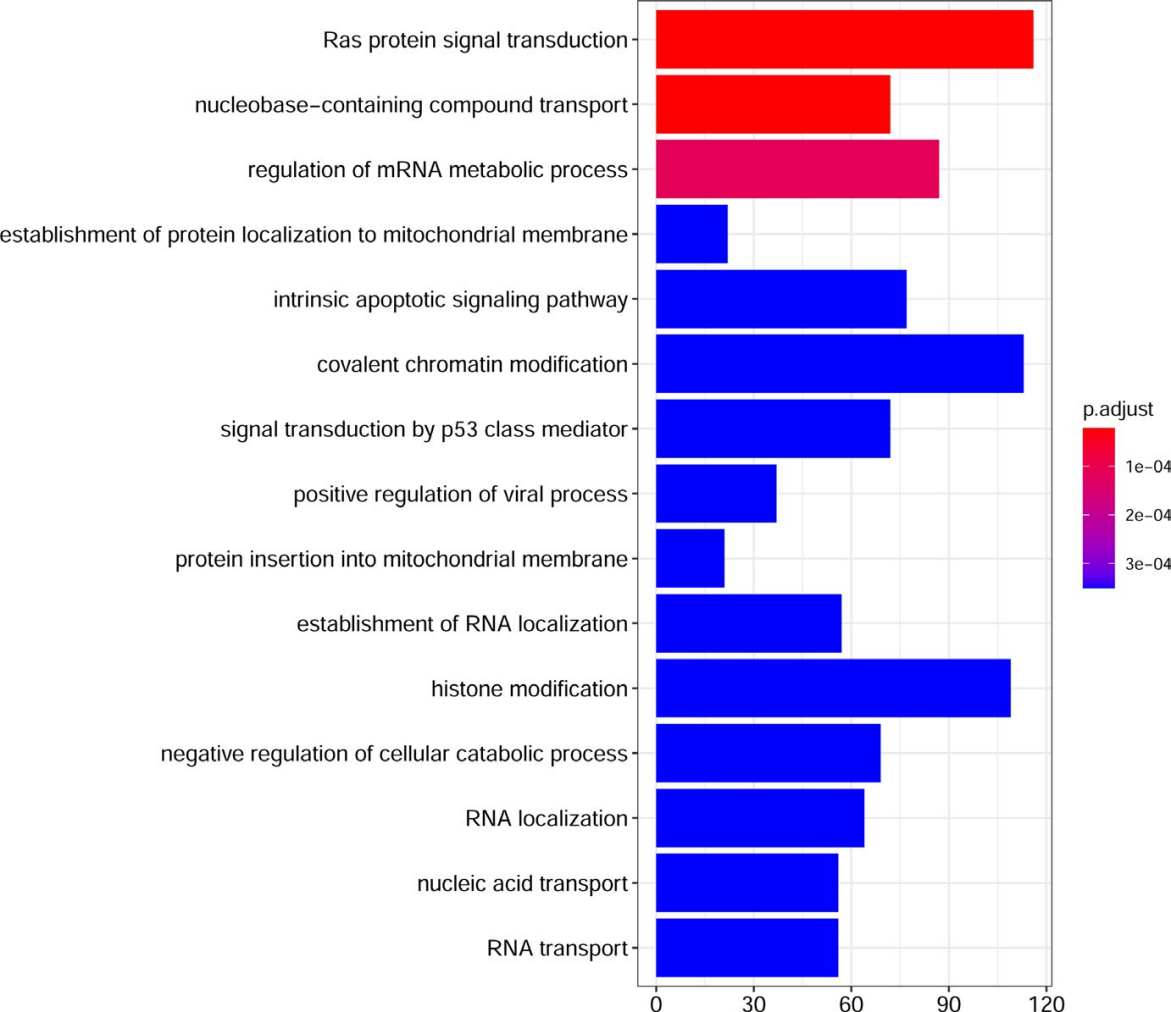
The top 15 biological processes and pathways with the most significant *p*‐values. The *x*‐axis represents the number of target genes involved in the pathway

We used circInteractome to predict the targeted miRNA of hsa_circ_0016148 and found a total of nine targeted miRNAs (hsa‐miR‐31‐3p, hsa‐miR‐31‐5p, hsa‐miR‐623, hsa‐miR‐634, hsa‐miR‐658, hsa‐miR‐1229‐3p, hsa‐miR‐1229‐5p, hsa‐miR‐1304‐3p, and hsa‐miR‐1304‐5p). Then, the target genes of these nine miRNAs were predicted from the TARBASE v.8 and miRTARBASE databases. Based on the above miRNAs and mRNA, the hsa_circ_0016148‐miRNA‐mRNA interaction network was constructed by Cytoscape (Figure 5). Meanwhile, GO enrichment analysis was conducted by all target genes to explore the mechanisms of hsa_circ_0016148 involved in the development of HNSCC. The top 15 pathways with the most significant *P*‐values are shown in Figure 6.

## 4 DISCUSSION

Despite tremendous research efforts and huge improvements in a combination of surgery, chemotherapy, radiotherapy, immunotherapy, and molecular‐targeted agents for HNSCC, most patients are diagnosed with a locally advanced stage and have poor outcomes.^22^ Identifying reliable biomarkers for early diagnosis is a key step to improve prognosis and quality of life for HNSCC patients.^23^ Therefore, there is an urgent need to develop and verify an effective and specific biomarker to improve the prevention, diagnosis, and prognosis prediction for HNSCC. Recent functional studies have revealed many tissue‐specific and cell‐specific circRNAs and functionally characterized them as efficient biomarkers in human diseases, especially cancers.^24^ For example, hsa_circ_0065149 has been demonstrated to be an indicator for early gastric cancer screening and prognosis prediction.^25^ However, the mechanism and the clinical application value of circRNAs in HNSCC are still largely unknown.

Utilization of high‐throughput sequencing and bioinformatic analysis can be efficient strategies to identify aberrantly expressed circRNAs in cancers. In the current study, we focused our attention on hsa_circ_0016148, based upon the results of microarray analysis. Our findings represented that hsa_circ_0016148 expression levels were downregulated in HNSCC. Additionally, multiple studies clarified that numerous circRNAs were correlated with clinicopathological parameters. We also analyzed the correlation between hsa_circ_0016148 expression levels and clinicopathological features of HNSCC patients and found that hsa_circ_0016148 expression levels are significantly lower in advanced patients than in early‐stage patients with HNSCC, which may also support a role for hsa_circ_0016148 in preventing tumorigenesis in HNSCC. Nodal metastasis affects the survival rate of HNSCC patients, and a poorer prognosis is often given based on larger tumor size and amount of nodal metastasis.^26^ A recent study by Valero showed that the total number of metastatic lymph nodes is the strongest predictor of outcomes in oral cavity squamous cell carcinomas.^27^ Our study reveals that hsa_circ_0016148 expression levels are associated with lymphatic metastasis. This implies that downregulated hsa_circ_0016148 might play an important role in lymphatic metastasis and be a prognostic indicator for patients with HNSCC.

Typically, diagnosis of HNSCC patients depends on clinical symptoms from the primary site, such as long‐lasting dysphagia, persistent hoarseness, epistaxis, oral mucosa ulcers, and otalgia. Patients in whom the primary neoplastic site is the oral cavity, upper glottis, or nasopharynx often present with cervical lymph node metastasis as their first presenting sign.^28^ Additionally, conventional tumor‐related blood biomarkers, such as carcinoembryonic antigen (CEA) and carbohydrate antigens 19–9 (CA19–9), do not have satisfactory sensitivity for HNSCC detection, especially in the early stages.^29^ Therefore, due to non‐specific symptoms in the early stages and lack of effective diagnostic biomarkers for HNSCC, the majority of HNSCC patients are diagnosed at an advanced stage (III and IV), at which point survival rates are low relative to those for early stages (I and II).^30^ Due to the stability, tissue specificity, and the spatiotemporal specificity of circRNA, it could be a novel biomarker in early diagnosis of cancer patients.^31^, ^32^ In this study, we also calculated ROC curves to assess the value of hsa_circ_0016148 in HNSCC diagnosis. The higher AUC value indicates the better diagnostic capability.^33^ In the current research, we demonstrated that hsa_circ_0016148 can distinguish HNSCC patients from normal individuals with an AUC value of 0.912. Meanwhile, it is worth noting that the specificity and sensitivity of hsa_circ_0016148 were near‐perfect at a suitable cut‐off point, suggesting that hsa_circ_0016148 can serve as an effective diagnostic marker for HNSCC. Increasing evidence shows that circRNAs can serve as prognostic markers for cancers.^34^ In our study, Kaplan–Meier plots indicate that low levels of hsa_circ_0016148 are significantly associated with a reduced overall survival rate for HNSCC. Additionally, Cox regression analysis confirmed that low‐expression levels of hsa_circ_0016148 is an independent poor predictive factor for overall survival of HNSCC patients. All above findings support the view that hsa_circ_0016148 expression levels could be a biomarker for determining the prognosis of HNSCC and tailoring personalization treatment. However, because of our relatively small sample size, further studies with large sample size and diverse population are needed to verify our results.

circRNAs can function as miRNA sponges to specifically bind miRNAs. In consequence, they can relieve the inhibitory effects of miRNA on downstream target genes, thus upregulating the expression levels of the target genes and forming various competing endogenous RNA (ceRNA) networks.^35^ Previous studies reveal that circRNAs are involved in regulation of several cancers as miRNA sponges, including HNSCC. Wang et al.^36^ revealed that circRNA_103862 promotes the proliferation and invasion of laryngeal squamous cell carcinoma through targeting the miR‐493‐5p/GOLM1 axis, and it might serve as a prognostic indicator and anti‐cancer target for laryngeal squamous cell carcinoma. Hao et al.^37^ found that upregulated circ‐0000495 mediates overexpression of TROP2 by sponging miR‐488‐3p to promote tumor progression and is associated with patient survival in HNSCC. Here, we explored the potential biological function of hsa_circ_0016148 in HNSCC tumorigenesis through bioinformatics analysis by constructing a hsa_circ_0016148‐miRNA‐mRNA interaction network. We found that hsa_circ_0016148 can bind nine miRNAs (hsa‐miR‐31‐3p, hsa‐miR‐31‐5p, hsa‐miR‐623, hsa‐miR‐634, hsa‐miR‐658, hsa‐miR‐1229‐3p, hsa‐miR‐1229‐5p, hsa‐miR‐1304‐3p, and hsa‐miR‐1304‐5p) to regulate the downstream target genes, which are associated with multiple key biological processes and cancer‐related signal pathways, such as Ras protein signal transduction, regulation of mRNA metabolic process, intrinsic apoptotic signaling pathway, and signal transduction by p53 class mediator. These findings give us a hint that hsa_circ_0016148 may work through hsa_circ_0016148‐miRNA‐mRNA to regulate numerous pathophysiological processes associated with HNSCC tumorigenesis and progression, a conjecture which should be validated by rigorous in vitro and in vivo experiments in future.

In conclusion, our research revealed that hsa_circ_0016148 is remarkably downregulated in HNSCC and may play an important role in HNSCC tumorigenesis via the hsa_circ_0016148‐miRNA‐mRNA axis. We also discovered that hsa_circ_0016148 is an effective biomarker for the diagnosis of HNSCC with a high degree of accuracy, specificity, and sensitivity. Additionally, our findings supported hsa_circ_0005986 as an independent unfavorable factor for overall survival in HNSCC patients.

## CONFLICTS OF INTEREST

The authors declare that there is no conflict of interest regarding the publication of this article.

## AUTHOR CONTRIBUTIONS

Liuqian Wang and Dong Ye wrote the manuscript. Zhisen Shen reviewed and edited the manuscript. Both authors have read and approved the final manuscript and take responsibility for its integrity.

## Data Availability

The data used to support the findings of this study are available from the corresponding author upon request.
